# VCAM‐1 upregulation accompanies muscle remodeling following resistance‐type exercise in Snell dwarf (*Pit1^dw/dw^*) mice

**DOI:** 10.1111/acel.12816

**Published:** 2018-07-10

**Authors:** Erik P. Rader, Marshall A. Naimo, James Ensey, Brent A. Baker

**Affiliations:** ^1^ Centers for Disease Control and Prevention National Institute for Occupational Safety and Health Morgantown West Virginia; ^2^ Division of Exercise Physiology West Virginia School of Medicine Morgantown West Virginia

**Keywords:** agonist muscles, antagonist muscles, dorsiflexor muscles, plantarflexor muscles, skeletal muscle, stretch‐shortening contractions

## Abstract

Snell dwarf mice (*Pit1^dw/dw^*) exhibit deficiencies in growth hormone, prolactin, and thyroid stimulating hormone. Besides being an experimental model of hypopituitarism, these mice are long‐lived (>40% lifespan extension) and utilized as a model of slowed/delayed aging. Whether this longevity is accompanied by a compromised quality of life in terms of muscular performance has not yet been characterized. In this study, we investigated nontrained and trained muscles 1 month following a general validated resistance‐type exercise protocol in 3‐month‐old Snell dwarf mice and control littermates. Nontrained Snell dwarf gastrocnemius muscles exhibited a 1.3‐fold greater muscle mass to body weight ratio than control values although muscle quality, maximum isometric torque normalized to muscle mass, and fatigue recovery were compromised. For control mice, training increased isometric torque (17%) without altering muscle mass. For Snell dwarf mice, isometric torque was unaltered by training despite decreased muscle mass that rendered muscle mass to body weight ratio comparable to control values. Muscle quality and fatigue recovery improved twofold and threefold, respectively, for Snell dwarf mice. This accompanied a fourfold increase in levels of vascular cell adhesion molecule‐1 (VCAM‐1), a mediator of progenitor cell recruitment, and muscle remodeling in the form of increased number of central nuclei, additional muscle fibers per unit area, and altered fiber type distribution. These results reveal a trade‐off between muscle quality and longevity in the context of anterior pituitary hormone deficiency and that resistance‐type training can diminish this trade‐off by improving muscle quality concomitant with VCAM‐1 upregulation and muscle remodeling.

## 1 INTRODUCTION

Hypopituitary mutant dwarf mice with combined anterior pituitary hormone deficiencies of growth hormone, thyrotropin, and prolactin are valuable genetic models for the investigation of both congenital hypopituitarism and lifespan extension (Bartke & Darcy, 2017). The Snell dwarf mouse, first described in 1929, is one such genetic model with a recessive mutation in *Pit1 (Pou1f1)*, an anterior pituitary transcriptional factor (Snell, 1929). *PIT1* is among the most common genes mutated in genetic cases of patients with combined pituitary hormone deficiencies with more than 30 distinct *PIT1* mutations identified to date (Stieg, Renner, Stalla, & Kopczak, 2017; Takagi et al., 2017). Such hypopituitarism results as a consequence of compromised anterior pituitary development, and clinical manifestation includes several various forms of muted development overall and short stature (Lee et al., 2011). Studies regarding the *Pit1* mutation in Snell dwarf mice have also demonstrated remarkable lifespan extension of >40% relative to littermates (Flurkey, Papaconstantinou, & Harrison, 2002; Flurkey, Papaconstantinou, Miller, & Harrison, 2001). Concomitant with this longevity is an apparent delay in aging in terms of data regarding T‐cell function, collagen cross‐linking, incidence of cataracts, resistance to cancer, and kidney disease (Alderman et al., 2009; Flurkey et al., 2001; Vergara, Smith‐Wheelock, Harper, Sigler, & Miller, 2004).

Despite enduring scientific interest in Snell dwarf mice, whether hypopituitary‐induced longevity in these mice compromises quality of life has not been fully tested especially in regard to skeletal muscle performance. Such data, in a limited manner, have been addressed for the closely related long‐lived Ames Dwarf mutant (*Prop1^df/^^df^*) mice which also exhibit deficiencies in growth hormone, thyrotropin, and prolactin (Brown‐Borg, Borg, Meliska, & Bartke, 1996). For these mice, percent lean body mass values were determined to be comparable (at 2 months of age) or increased (4.5–6 months of age) relative to age‐matched controls (Heiman, Tinsley, Mattison, Hauck, & Bartke, 2003). In addition, in a wire hang test administered at middle age to old age (19‐ to 30‐month‐old), Ames dwarf mice were able to maintain grip for a longer duration than age‐matched controls demonstrating prevention of age‐related neuromusculoskeletal decline in a distinct test (Arum, Rasche, Rickman, & Bartke, 2013). Although these studies provide some insight into the effects of anterior pituitary hormone deficiency, further evaluation of skeletal muscle mass specifically and muscle performance under high activation especially at young age is required for a more complete characterization. Furthermore, the response to mechanical loading in the form of resistance‐type exercise training has not been tested.

The purpose of this study was to investigate skeletal muscle of young (3‐month‐old) Snell dwarf mice and their littermate controls at the onset and completion of 1 month of resistance training with stretch‐shortening contractions (SSCs), contractions typical during resistance‐type exercise training (Vaczi et al., 2014, 2011). The SSC protocol consisted of 80 maximally activated SSCs (8 sets with 10 repetitions per set) and has been validated repeatedly to increase performance for muscles of rats and mice (Cutlip et al., 2006; Rader et al., 2016; Rader, Naimo, Ensey, & Baker, 2017). Because of the potential for opposing training‐induced results for agonist vs. antagonist muscles, the agonist gastrocnemius and antagonist tibialis anterior (TA) muscles were evaluated (Rader et al., 2017). In conclusion, to determine whether muscle remodeling involving vascular endothelial growth factor (VEGF) and VCAM‐1, a mediator of progenitor cell recruitment following exercise, was possibly influential in the response, levels of these proteins were assessed (Stromberg, Rullman, Jansson, & Gustafsson, 2017). The results provide further insight into the role of anterior pituitary hormones on muscle mass and quality in nontrained and resistance‐type trained agonist muscles and the potential influence of VCAM‐1 as compensatory. The findings also confirm the concern regarding antagonist muscle atrophy following isolated muscle training for specific muscles.

## 2 RESULTS

### 2.1 Muscles of Snell dwarf mice were large for their body weight yet exhibited substantial weakness evident by low muscle quality and compromised recovery from fatigue

**Figure 1:**
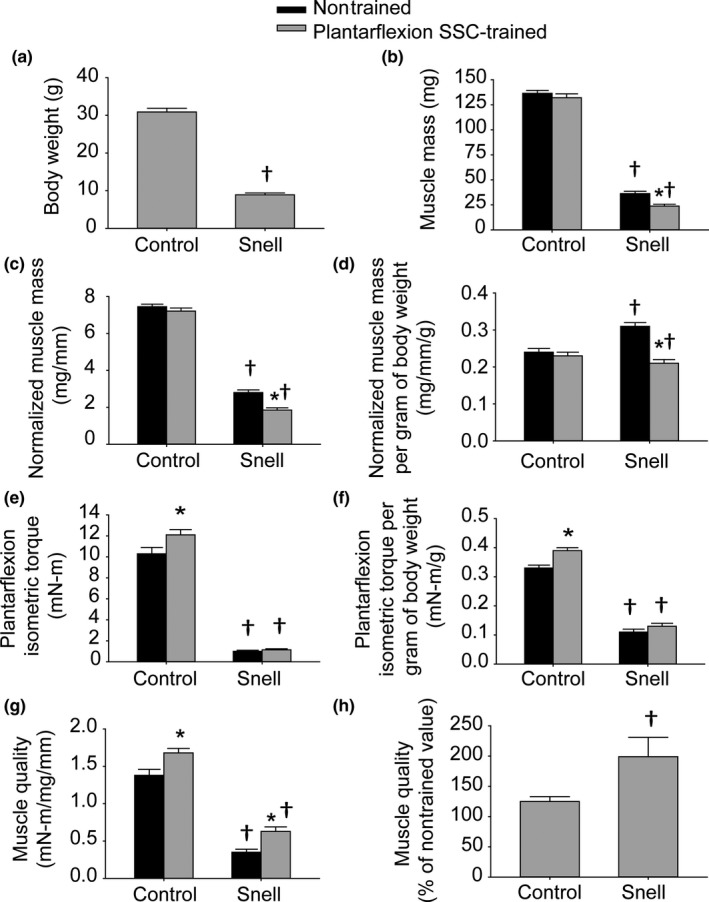
Plantarflexion SSC training reduces the deficit in GTN muscle quality inherent with the *Pit1* mutation. Values were determined for (a) body weight, (b) GTN muscle mass, (c) GTN muscle mass normalized to tibial length, (d) GTN normalized muscle mass per gram of body weight, (e) maximum plantarflexion isometric torque, (f) maximum plantarflexion isometric torque per gram of body weight, (g) plantarflexor muscle quality, and (h) plantarflexor muscle quality following training expressed as a percentage of nontrained values. Sample sizes were *N* = 7 to 11 per group. Values are means ± *SE*. *Different from nontrained value; ^†^Different from control value, *p < *0.05

**Figure 2:**
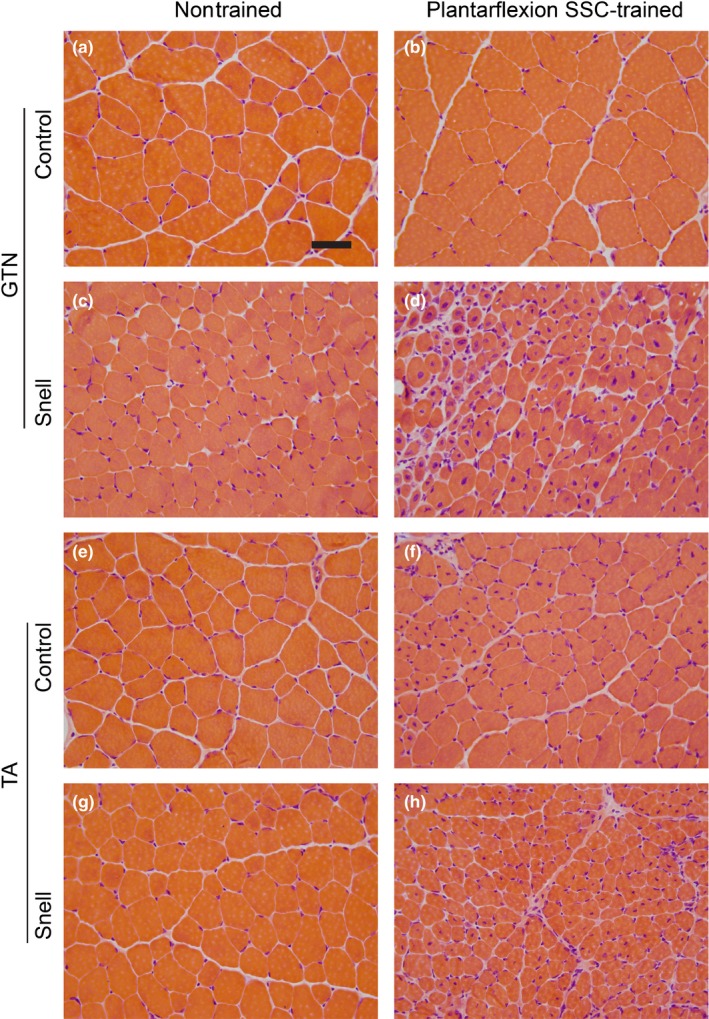
Transverse sections of nontrained and plantarflexion SSC‐trained muscles stained with hematoxylin and eosin for control (a–d) and Snell dwarf mice (e–h). Scale bar = 50 µm

**Figure 3:**
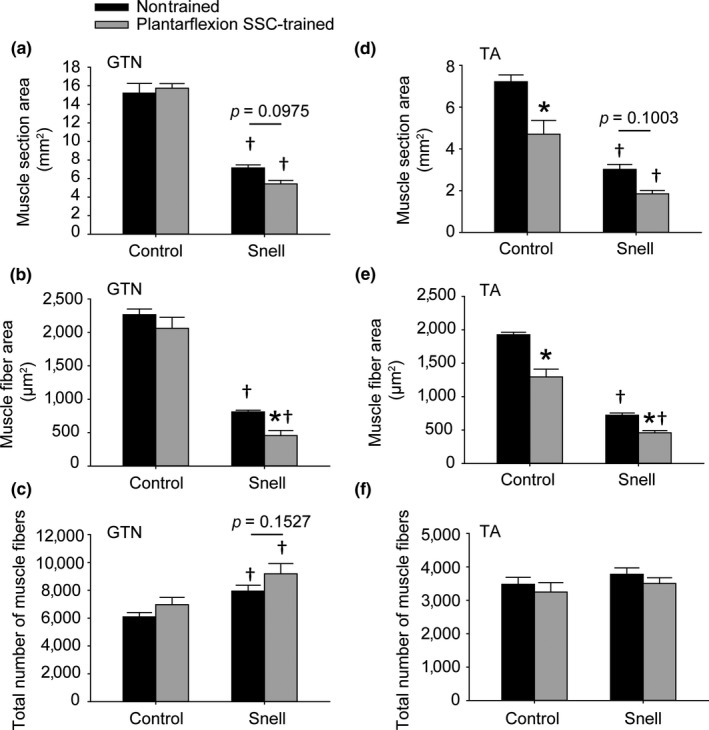
Muscles of Snell dwarf mice exhibited a high number of small muscle fibers although exposure to plantarflexion SSC training exaggerated this phenotype in GTN muscles (a–c). Following such training, TA muscle fibers atrophied with no indication of increasing in number regardless of mouse genotype (d–f). A total number of muscle fibers were determined per muscle section. Sample sizes were *N* = 5 to 6 per group. Values are means ± *SE*. *Different from nontrained value; ^†^Different from control value, *p < *0.05

Body weights for Snell dwarf mice were 29% of control values (Figure 1a). Tibial lengths for Snell dwarf mice (12.9 ± 0.2 mm for nontrained limb and 12.8 ± 0.2 mm for trained limb) were 70% of control values (18.3 ± 0.2 mm for nontrained limb and 18.3 ± 0.2 mm for trained limb), *p* < 0.0001 for Snell dwarf vs. control mice. Muscle mass was normalized to tibial length as an estimate of girth (Figure 1b,c). For nontrained muscles, Snell dwarf normalized muscle masses were 42% of control values for TA muscles (1.16 ± 0.05 mg/mm vs. 2.77 ± 0.03 mg/mm, *p* < 0.0001) and 38% of control values for GTN muscles (Figure 1c). The decreased muscle mass in the nontrained state for Snell dwarf mice was attributable to decreased muscle fiber size (Figures 2c and 3a–c). When expressed per gram body weight, nontrained normalized GTN muscle mass was elevated for Snell dwarf mice demonstrating that the muscles of Snell dwarf mice were large for their size (Figure 1d). This finding was confirmed for nontrained TA muscles with normalized muscle mass per gram body weight values for Snell dwarf vs. control mice of 13.1 ± 4.0 mg mm^−1^ g^−1^ vs. 9.0 ± 2.2 mg mm^−1^ g^−1^, respectively, *p* < 0.0001. However, nontrained muscles of Snell dwarf mice were weak in terms of both plantarflexion peak dynamic torque (1.5 ± 0.2 mN‐m vs. 13.9 ± 0.8 mN‐m, Snell dwarf vs. control, *p* < 0.0001) and maximum isometric torque, even after expressing per gram body weight (Figure 1e,f). Therefore, plantarflexor muscle quality of nontrained Snell dwarf muscle was 25% that of controls (Figure 1g). Fatigue measures were also assessed in the nontrained state, and a compromised recovery from fatigue was observed for Snell dwarf mice (Supporting Information Figure S1A–E). By 5 min following an SSC session, control muscles recovered 47 ± 4% of pre‐SSCs isometric torque (Supporting Information Figure S1E). Meanwhile, Snell dwarf muscles only recovered 9% ± 2% (Supporting Information Figure S1E).

### 2.2 Plantarflexion training induced an exceptional improvement in Snell dwarf plantarflexor muscle quality and fatigue recovery

For control mice, plantarflexion SSC training did not alter GTN muscle mass (Figure 1b–d). Peak dynamic torque (13.9 ± 0.8 mN‐m to 17.0 ± 0.8 mN‐m, *p* = 0.002), maximum isometric torque, and muscle quality increased by 20% (Figure 1e–h). No overt alteration in muscle tissue composition was observed in GTN muscle sections upon training (Figure 2a,b and Table 1). For Snell dwarf mice, absolute performance measures—peak dynamic torque (1.5 ± 0.2 mN‐m and 1.8 ± 0.1 mN‐m, nontrained vs. trained) and maximum isometric torque—were maintained following plantarflexion SSC training (Figure 1e,f). It is an interesting fact this maintenance of performance occurred despite a decrease in GTN muscle mass so that normalized muscle mass per gram body weight values became comparable to control values (Figure 1b–d). Therefore, muscle quality increased exceptionally (twofold) for Snell dwarf mice (Figure 1h). For both groups of mice, SSC training improved the absolute torque values generated during the SSC session without increasing fatigue rate (Supporting Information Figure S1A–D). Furthermore, fatigue recovery was enhanced by training especially for Snell dwarf mice—a threefold increase (Supporting Information Figure S1E). These training‐induced performance outcomes in Snell dwarf mice were accompanied by alterations in muscle composition apparent with hematoxylin and eosin staining (Figure 2). Quantitative morphology analysis demonstrated an increase in GTN muscle interstitium (Table 1). Training induced a decrease in GTN muscle fiber area by 44 ± 8% and a GTN muscle fiber number value of 9,180 ± 734 vs. 7,937 ± 425 (trained vs. nontrained, *p* = 0.1527; Figures 2d and 3a–c). Muscle tissue remodeling was also evident by an increased percentage of centrally nucleated muscle fibers (Table 1).

**Table 1:** Quantitative morphology for the composition of muscles of control and Snell dwarf mice exposed to plantarflexion SSC training

	Nontrained	Plantarflexion SSC‐trained
GTN		
Control		
Nondegenerative muscle fibers (%)	90.8 ± 0.8	89.3 ± 1.0
Degenerative muscle fibers (%)	0.0 ± 0.0	0.0 ± 0.0
Noncellular interstitium (%)	8.8 ± 0.8	10.1 ± 1.0
Cellular interstitium (%)	0.4 ± 0.1	0.6 ± 0.2
Centrally nucleated fibers (%)	1.5 ± 0.5	6.0 ± 1.9
Snell		
Nondegenerative muscle fibers (%)	89.7 ± 0.8	73.2 ± 6.7^a^, ^b^
Degenerative muscle fibers (%)	0.0 ± 0.0	0.1 ± 0.1
Noncellular interstitium (%)	9.6 ± 0.8	22.1 ± 5.3^a^, ^b^
Cellular interstitium (%)	0.7 ± 0.1	4.6 ± 1.5^a^, ^b^
Centrally nucleated fibers (%)	1.3 ± 0.4	36.0 ± 4.8^a^, ^b^
TA		
Control		
Nondegenerative muscle fibers (%)	92.4 ± 0.5	90.3 ± 0.9
Degenerative muscle fibers (%)	0.0 ± 0.0	0.0 ± 0.0
Noncellular interstitium (%)	7.1 ± 0.4	8.7 ± 0.8
Cellular interstitium (%)	0.5 ± 0.2	1.0 ± 0.1
Centrally nucleated fibers (%)	2.2 ± 0.7	8.5 ± 3.5
Snell		
Nondegenerative muscle fibers (%)	90.6 ± 0.8	87.4 ± 0.9^a^, ^b^
Degenerative muscle fibers (%)	0.0 ± 0.0	0.0 ± 0.0
Noncellular interstitium (%)	8.2 ± 1.1	11.0 ± 0.9^a^, ^b^
Cellular interstitium (%)	1.1 ± 0.1	1.6 ± 0.3^a^, ^b^
Centrally nucleated fibers (%)	1.3 ± 0.4	23.1 ± 8.9^a^, ^b^

Note

Values are means ± *SE*. Percentage refers to percentage of tissue. Sample sizes were *N* = 5 to 6 per group.

a Different from nontrained muscle.

b Different from control muscle, *p* < 0.05.

### 2.3 Antagonist TA muscles atrophied for both control and Snell dwarf mice following the plantarflexion training

Plantarflexion SSC training induced a ~35% decrease in the antagonist TA muscle mass for both control (2.77 ± 0.03 mg/mm to 1.88 ± 0.03 mg/mm, *p* < 0.0001) and Snell dwarf mice (1.16 ± 0.05 mg/mm to 0.72 ± 0.04 mg/mm, *p* = 0.0006). This loss in muscle tissue was accompanied by alterations in muscle composition as evident by a training main effect of increased percentage of tissue of interstitium and presence of centrally nucleated muscle fibers which reached significance for the Snell dwarf mice (Figure 2e–h, Table 1). Muscle fiber size decreased by ~35% for both groups of mice and, unlike the agonist GTN muscle, no indication of increased muscle fiber number was observed (Figure 3d–f). Protein levels for six cytokines were investigated, interferon gamma, interleukin‐6, interleukin‐10, interleukin‐12, interleukin‐17, and tumor necrosis factor alpha, and no training‐induced change was observed (Table 1 and Supporting Information Table S1). These results suggest that the TA atrophy was not accompanied by a response in these cytokines.

### 2.4 Training‐induced alterations in VCAM‐1 and fiber type distribution indicative of robust GTN muscle remodeling correlated with improved performance for Snell dwarf mice

**Figure 4:**
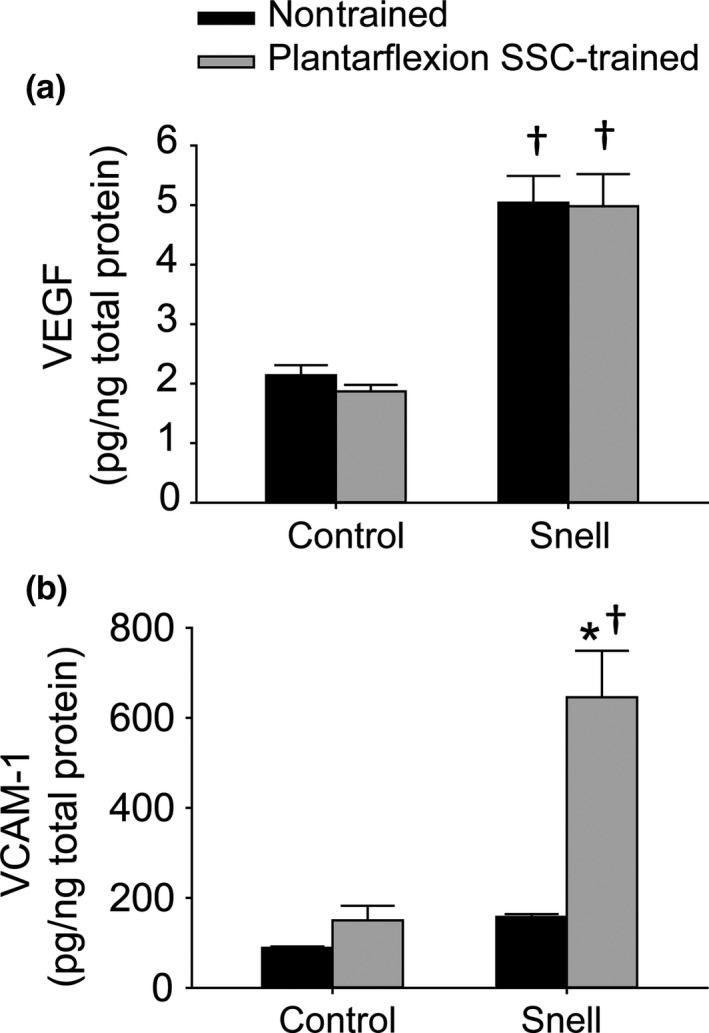
VEGF and VCAM‐1 protein levels within GTN muscle homogenates following plantarflexion SSC training. Sample sizes were *N* = 7 to 10 per group. Values are means ± *SE*. *Different from nontrained value; ^†^Different from control value, *p < *0.05

**Figure 5:**
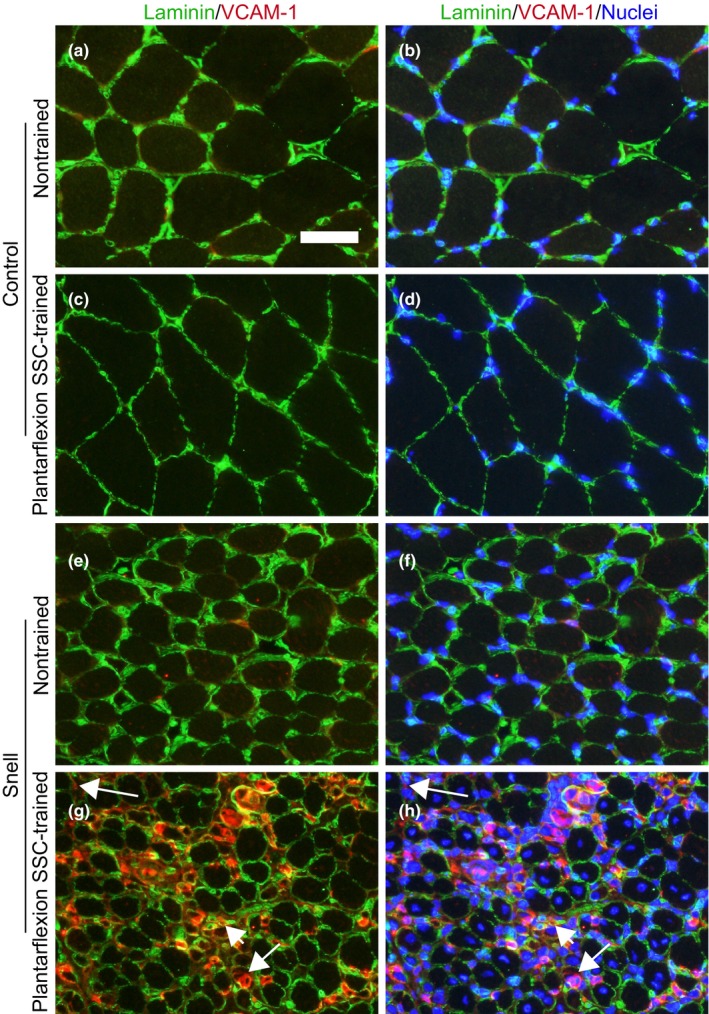
Immunofluorescence in nontrained and plantarflexion SSC‐trained GTN muscles for the combinations of VCAM‐1 (red) and laminin (green) (a, c, e, and g) and VCAM‐1 (red), laminin (green), and nuclei (blue) (b, d, f, and h). Overt VCAM‐1 immunostaining was apparent within nodes (e.g., short arrow), muscle fibers (e.g., medium length arrow), and interstitium (e.g., long arrow) in GTN muscles of Snell dwarf mice following SSC training. Scale bar = 50 µm

**Figure 6:**
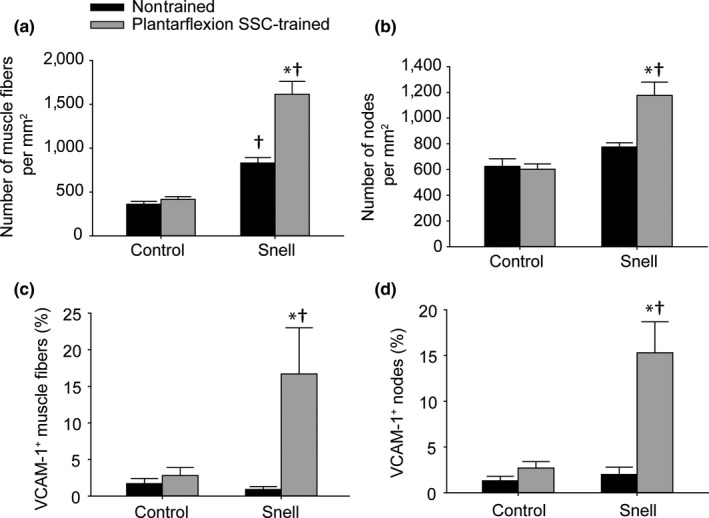
The SSC‐induced increase in muscle fibers nodes per unit area and for Snell dwarf GTN muscles was accompanied by increased VCAM‐1^+^ staining. Total number of (a) muscle fibers and (b) nodes per muscle section area. Percentage of (c) muscle fibers and (d) nodes with VCAM‐1^+^ staining. Sample sizes were *N* = 5 to 6 per group. Values are means ± *SE*. *Different from nontrained value; ^†^Different from control value, *p < *0.05

To investigate further the remodeling apparent in trained GTN muscles especially for Snell dwarf mice, the protein levels of VEGF and VCAM‐1 were evaluated. For nontrained muscles, VEGF was elevated twofold for Snell dwarf mice relative to control mice (Figure 4a). Training had no effect on VEGF levels. However, training had a pronounced effect (i.e., fourfold increase) on VCAM‐1 levels specifically for Snell dwarf mice (Figure 4b). Immunofluorescence labeling for laminin, nuclei, and VCAM‐1 was undertaken to determine the distribution of VCAM‐1 (Figure 5). The twofold increase in the number of muscle fibers per unit area observed for trained Snell dwarf muscle was accompanied by a comparable increase in a number of nodes (i.e., anatomical features encircled by laminin and adjacent to muscle fibers—characteristics indicative of capillaries) per unit area (Figure 6a,b). Furthermore, these increases in muscle fibers and nodes coincided with increases in the incidence of VCAM‐1^+^ staining in these features (Figure 6c,d). Close inspection of the immunostaining revealed that VCAM‐1 staining tended to circle or cluster adjacent to nuclei within muscle fibers, nodes, and the interstitium (Figure 5). Therefore, an analysis of the nuclei was undertaken which demonstrated an overall increase in nuclei number per unit area for trained Snell dwarf mice in the various tissue compartments analyzed (Table 2). Concomitant with this training‐induced increase in nuclei number was an increase in percentage of VCAM‐1 staining associated with these nuclei (Table 2).

**Table 2:** Quantitative analysis of nuclei for association with VCAM‐1 staining in cross sections of GTN muscles

	Nontrained	Plantarflexion SSC‐trained
Control		
Total number per mm^2^		
Muscle fiber peripheral nuclei	471 ± 60	499 ± 28
Muscle fiber central nuclei	5 ± 2	70 ± 24
Nodal nuclei	338 ± 35	304 ± 29
Interstitial nuclei	88 ± 22	136 ± 28
VCAM‐1 associated (%)		
Muscle fiber peripheral nuclei	0.7 ± 0.7	0.5 ± 0.2
Muscle fiber central nuclei	0.0 ± 0.0	0.8 ± 0.8
Nodal nuclei	1.6 ± 0.7	2.6 ± 1.0
Interstitial nuclei	2.5 ± 1.8	10.8 ± 4.7
Snell		
Total number per mm^2^		
Muscle fiber peripheral nuclei	646 ± 55	916 ± 231
Muscle fiber central nuclei	11 ± 5	694 ± 103^a^, ^b^
Nodal nuclei	354 ± 28	542 ± 57^a^, ^b^
Interstitial nuclei	103 ± 47	737 ± 179^a^, ^b^
VCAM‐1 associated (%)		
Muscle fiber peripheral nuclei	0.9 ± 0.3	11.2 ± 3.9^a^, ^b^
Muscle fiber central nuclei	0.0 ± 0.0	1.3 ± 0.8
Nodal nuclei	2.4 ± 1.1	16.5 ± 6.4
Interstitial nuclei	0.6 ± 0.6	19.0 ± 5.4^a^

Note

Values are means ± *SE*. Total number per mm^2^ refers to total number of both VCAM‐1 associated and nonassociated nuclei. Percentage refers to percentage of total number associated with VCAM‐1 staining. Sample sizes were *N* = 5 to 6 per group.

a Different from nontrained value.

b Different from control value, *p* < 0.05.

Stretch‐shortening contraction training‐induced GTN muscle remodeling for Snell dwarf mice also extended to fiber type alterations consistent with a shift to a more oxidative phenotype as apparent in immunofluorescence labeling (Supporting Information Figure S2). Type IIb fibers decreased in cross‐sectional area to 40% of nontrained area and percentage of muscle tissue composed of type IIb fibers decreased to 55% of control value (Supporting Information Figure S3A,C). Meanwhile, the number of type IIx fibers per unit area increased by sevenfold and percentage of IIx muscle fibers (relative to a total number of muscle fibers) increased by fourfold (Supporting Information Figure S3C,D). This altered fiber type distribution from type IIb to IIx fibers with training was confirmed by chi‐square analysis (Supporting Information Figure S3E,F). This SSC‐induced remodeling at the fiber type level for Snell dwarf mice was coordinated with VCAM‐1 distribution as suggested by the correlation between these factors (Supporting Information Fig. S4). Both high percentages of VCAM‐1^+^ muscle fibers and nodes were accompanied by a high percentage of type IIx fibers (Supporting Information Figure S4F,G). This extensive muscle remodeling evident by VCAM‐1 and fiber type distribution correlated with the improved fatigue recovery gained by Snell dwarf muscle following training (Supporting Information Figure S4H,I,J). Pearson product correlation analysis was also performed for Snell dwarf muscle quality vs. percentage VCAM‐1^+^ nodes (*r* = 0.584, *p* = 0.046), type IIx muscle fibers (*r* = 0.507, *p* = 0.092), and VCAM‐1^+^ muscle fibers (*r* = 0.485, *p* = 0.11). Overall, the findings indicated that the Snell dwarf muscle tissue remodeling impacted performance in general.

## 3 DISCUSSION

Hypopituitarism characterized by diminished pituitary gland function can result from multiple underlying causes with a prevalence of 45.5 cases per 100,000 internationally (Regal, Paramo, Sierra, & Garcia‐Mayor, 2001). Combined deficiencies of growth hormone, thyrotropin, and prolactin are typical in congenital hypopituitarism, a form of hypopituitarism in which mutation in *PIT1*is a common genetic cause (Stieg et al., 2017; Takagi et al., 2017). The present study characterizes in the Snell dwarf (*Pit1^dw/dw^*) mouse model, the impact of such combined deficiencies on skeletal muscle mass, function, and response to resistance‐type exercise training. The finding of a high muscle mass to body mass ratio concomitant with low muscle quality and compromised fatigue recovery for nontrained muscles of Snell dwarf mice demonstrates a distinct phenotype and that anterior pituitary hormones regulate these outcomes. The low muscle quality and fatigue recovery in the nontrained state demonstrated an instance of the trade‐off between vigor vs. longevity inherent in Snell dwarf mice (Bartke, Sun, & Longo, 2013). Following resistance‐type training, maximum static and dynamic performance of agonist GTN muscles improved by 20% for control mice although such measures were unaltered for Snell dwarf mice. Despite the lack of change in absolute strength with training, muscle quality and fatigue recovery capability improved. VCAM‐1 upregulation and muscle remodeling accompanied these functional improvements, thereby implicating progenitor cell recruitment as a mediator for these compensatory alterations. Although muscles of Snell dwarf mice did not increase maximal strength in absolute terms following training, the outcome of maintained performance output with smaller muscles is a positive response to training in terms of efficiency.

A high percent lean body mass with combined pituitary hormone deficiency in the nontrained state was previously demonstrated at young to middle age for Ames dwarf mice (Heiman et al., 2003). However, assessment of skeletal muscle mass and quality was not addressed in that study. The high muscle mass to body weight ratio in nontrained muscles of young Snell dwarf mice of the present study is consistent with the report regarding Ames dwarf mice (Heiman et al., 2003). The finding that muscle fiber area rather than number was decreased in Snell dwarf mice complements morphological measures in hearts of young Ames dwarf mice demonstrating that cardiomyocytes were 46% smaller than controls (Helms et al., 2010). This indicates a distinct role of anterior pituitary hormones in regulating striated muscle fiber size in general. The most striking result was the low muscle quality (1/4th that of control values) of this tissue, thereby resulting in diminished performance per unit body weight. Quantitative morphology measures indicate that the poor muscle quality was not a result of overt degeneration or decrease in muscle fiber number. Low muscle quality was possibly linked to the deficiency in growth hormone and, consequently, secondary deficiency in circulating insulin‐like growth factor 1 (IGF‐1)/insulin signaling. Muscle strength associates with genetic variation in *IGF1* in individuals, and patients with disrupted growth hormone/IGF‐1/insulin signaling tend to exhibit diminished muscle quality (Cuneo & Wallace, 2005; Cuneo, Salomon, Wiles, & Sonksen, 1990; Huuskonen et al., 2011).

The resistance‐type SSC training utilized in the present study improved muscle quality and fatigue recovery capability by exceptional magnitudes, twofold and threefold, respectively, in plantarflexor muscles of Snell dwarf mice. This was concomitant with a decrease in muscle fiber size coupled with an increase in muscle fiber number per unit area. Such an outcome of muscle fiber decreased size and increased number coupled with muscle quality gain was also observed for soleus muscles of Sprague Dawley rats following volitional resistance training (Rader et al., 2017). Mechanisms proposed in that study included features inherent to having a high density of small muscle fibers which could improve performance—for example, a diminished metabolic/diffusion gradient consequently potentially improving energetics and a high sarcolemmal to cytoplasmic volume ratio, thereby possibly improving lateral force transmission (Rader et al., 2017). Another morphological feature may also play a role in the present study—the increased density of laminin‐encircled features (nodes) indicative of capillaries adjacent to muscle fibers. Resistance training has been demonstrated previously to increase vascularization and, thereby, improve performance (Verdijk, Snijders, Holloway, Van Kranenburg, & Van Loon, 2016). In regard to the training‐induced improvement in fatigue recovery, in particular, remodeling of the fiber type distribution to a more oxidative phenotype likely had a direct impact on muscle function. Following exercise, cells such as monocytes and endothelial progenitor cells must traverse the endothelium for angiogenesis and skeletal muscle fiber remodeling. VCAM‐1 is a key mediator of this process (Stromberg et al., 2017). The finding that for Snell dwarf mice, VCAM‐1 increased fourfold for GTN muscles with training and was localized within muscle fibers and nodes to an increased extent implicates VCAM‐1 as a mediator of the remodeling and increased density (number per unit area) of these features. In an interesting manner, nuclei within muscle fibers, nodes, and the interstitium increased in trained Snell dwarf muscle in the absence of increased muscle fiber degeneration supporting the notion of a remodeling rather than necrotic process. An increased percentage of these nuclei associated with VCAM‐1 immunostaining was observed and suggested that these nuclei were possibly of circularity cell origin and contributing directly to the muscle tissue alterations. This finding complements earlier research regarding treadmill exercise in mice and cycling exercise in humans, demonstrating increased expression of adhesion molecules with increased levels of distinct circulatory cells and transmigration across the endothelium (Nunes‐Silva et al., 2014; Stromberg et al., 2017). The present study complements this research by extending this phenomenon to resistance‐type exercise training and as a potential compensatory mechanism when anterior pituitary hormones are deficient.

Although the training was beneficial in regard to muscle quality and fatigue recovery capacity for Snell dwarf mice, responsiveness was muted for absolute performance measures, maximum isometric torque, and peak dynamic torque. This demonstrated the trade‐off between growth vs. longevity with anterior pituitary hormone deficiency (Bartke et al., 2013; Sharples et al., 2015). Such a trade‐off is consistent with reports regarding IGF‐1, a key hormone regulated by growth hormone. Individuals with *IGF1* variants associated with high IGF‐1 blood levels gain more strength following resistance training (Hand et al., 2007; Kostek et al., 2005). Yet, genetic polymorphisms in the gene for IGF‐1 receptor, which result in decreased IGF‐1 plasma levels, associate with longevity (Bonafe et al., 2003; Suh et al., 2008). Although the responsiveness to resistance‐type exercise training may be muted, absolute performance gains may still be possible as evident by research regarding growth hormone deficient adults (Werlang Coelho et al., 2002). As demonstrated with previous research regarding aging, careful design and refinement of resistance‐type exercise training consisting of SSCs has great potential for benefiting muscle even for muscle in an anabolic‐compromised state (Rader & Baker, 2017; Rader et al., 2016). Exercise prescription must also account for effects on surrounding muscles as evident by the finding of 35% atrophy for the antagonist TA muscles of both control and Snell dwarf mice following training in the present study. The observation of antagonist muscle atrophy confirmed an earlier report regarding the same plantarflexion SSC training to C57BL/6 mice (Rader et al., 2017). This response was attributed to a possible neuromuscular imbalance imposed on antagonist non‐weight‐bearing muscles such as the TA muscle when training is isolated to weight‐bearing muscles (Rader et al., 2017). The present study establishes that this training‐induced muscle imbalance is independent of anterior pituitary hormone levels. Overall, our findings support the further refinement and utilization of balanced resistance‐type exercise training for benefiting skeletal muscle in cases of hypopituitarism.

## 4 MATERIALS AND METHODS

### 4.1 Animals

The young (3 months old at onset of training) male Snell dwarf (*Pit1^dw/dw^*) mice and their age‐matched normal‐sized control littermates evaluated for this study were F1 generation produced by bidirectional mating of DW/J *Pit1^dw/+^* (Jax# 000643) and B6.DW *Pit1^dw/+^* (Jax# 000772) mice. Data from littermate heterozygote (*Pit1^dw/+^*) and homozygote wild‐type (*Pit1^+/+^*) mice were pooled and characterized as controls. Snell dwarf mice and their littermate controls were provided NIH‐31 Open diet (Teklad 7917). At least one normal‐sized female littermate was housed with Snell dwarf mice to provide warmth. The mice were housed in an AAALAC‐accredited animal quarters. All animal procedures were approved by the Animal Care and Use Committee at the National Institute for Occupational Safety and Health in Morgantown, WV.

### 4.2 Plantarflexion SSC training

Plantarflexor muscles were exposed to SSC training based on a previous procedure demonstrated to induce muscle mass and performance gains in young C57BL/6 J mice (Rader et al., 2017). For each training session, the mouse was anesthetized with isoflurane gas, placed in dorsal recumbency on a heated table, and the left foot secured to a footplate of a dual mode muscle lever system (Whole Mouse Test System, 1300A; Aurora Scientific). Platinum electrodes were placed subcutaneously to activate the tibial nerve, and muscle stimulation was set at parameters (8‐V magnitude, 0.2‐ms pulse duration, and 150‐Hz frequency) for maximal contraction. Prior to the 80 SSC training, static and dynamic performance was assessed. A single maximal isometric contraction with the ankle at 90° (angle between tibia and foot) was utilized for static performance, and a single SSC test was utilized for dynamic performance consisting of an isometric contraction for 200 ms at a 110° ankle angle followed by rotation to 70° at 500° per second and back to 110° at 500° per second while continuing activation for 200 ms.

At 2 min following the single SSC test, the training session was administered. Each training session consisted of 8 sets (2‐min intervals between sets) with 10 SSCs per set (3‐s intervals between SSCs). For each SSC, the muscles were maximally activated at ankle angle 90° for 100 ms, rotated to 70° at 60°/s, returned to 90° at 60°/s, and deactivated 100 ms later. Chronic exposure to SSCs with a velocity of 60°/s induces adaptation without overt muscle inflammation and degeneration in young wild‐type rodents days to weeks into training (Baker, Hollander, Kashon, & Cutlip, 2010; Baker, Hollander, Mercer, Kashon, & Cutlip, 2008; Cutlip et al., 2006; Rader et al., 2017). At 5 min following the 80 SSCs, a maximal isometric tetanic contraction was measured and compared with pre‐80 SSC exposure values to determine recovery from fatigue (Rader et al., 2017). Training sessions were administered for 4 weeks 2–3 days per week. Performance measures for sessions during the first and last week were averaged to determine initial nontrained and trained values, respectively. At 72 hr after the final SSC exposure, hindlimb muscles were removed, weighed, and the tibia length recorded. For trained muscles and contralateral nontrained muscles, muscle mass was divided by tibia length to determine normalized muscle mass. Muscle quality was determined by dividing maximal isometric torque by normalized muscle mass. The mid‐belly portion of the muscles was covered with tissue freezing media (Tissue‐Tek, Sakura Finetek) and frozen in cold isopentane (−80°C) for quantitative morphology and immunofluorescence. A portion of the remaining tissue was allocated for ELISA.

### 4.3 Quantitative morphology

Cryosections (12‐µm‐thick) were stained with hematoxylin and eosin and analyzed using a standardized stereological method (Baker et al., 2006; Rader et al., 2017, 2016). With the investigator blinded to sample identification, two sites were identified one right and one left of the section midline. At each site, a column of five fields (at 40× magnification) or the maximum possible fields without field overlap were analyzed. For each field, points of a 121‐point 11‐line overlay graticule (0.04 mm^2^ square with 100 divisions) were evaluated. Each point was identified as overlaying a muscle fiber (degenerative or nondegenerative) or interstitium (cellular or noncellular). Loss of contact with surrounding fibers, interdigitation of the sarcolemma by cellular infiltrates, and internalization of cellular infiltrates characterized degenerative muscle fibers (Baker et al., 2006). A point overlying a nucleus in between muscle fibers was characterized as cellular interstitium although a point between muscle fibers overlying a region lacking nuclei was considered noncellular interstitium. Percent of muscle tissue comprised of nondegenerative muscle fibers, degenerative muscle fibers, centrally nucleated muscle fibers, cellular interstitium, or noncellular interstium were calculated as the percentage of points which overlaid each type of tissue relative to the total number of points. Furthermore, all muscle fibers in which the topmost part of the fiber was within the graticule boundary were counted to determine the number of muscle fibers per unit cross‐sectional area. Mean muscle fiber area (µm^2^) was determined by dividing the percent of tissue comprised of muscle fibers by fiber number per unit area. Total number of muscle fibers was estimated by multiplying the area of the entire muscle section by the percent muscle tissue comprised of muscle fibers and the fiber number per unit area.

### 4.4 Immunofluorescence

Transverse cryosections of gastrocnemius (GTN) muscles were analyzed for myosin heavy chain (MHC) staining using a previously described method (Rader et al., 2017). Sections were blocked (10% goat serum in PBS) for 1 hr at room temperature and incubated in a primary antibody cocktail overnight at 4°C—antibodies against MHC I (BA‐F8; 1:10), MHC IIa (SC‐71; 1:200), MHC IIb (BF‐F3; 1:200), and laminin (L9393; 1:400). Sections were then exposed to a cocktail of secondary antibodies (Alexa Fluor from Life Technologies) for 2 hr at room temperature—350 IgG2b goat anti‐mouse (A21140; 1:250), 594 IgG1 goat anti‐mouse (A21125; 1:100), 488 IgM goat anti‐mouse (A21042; 1:500), and 488 IgG goat anti‐rabbit (A11008; 1:500). Fiber type analysis was performed by a standardized stereological method with the investigator blinded to sample identification (Rader et al., 2017). Nonoverlapping images were captured at the sites of most area in both the lateral and medial regions of the muscle section. An overlay graticule (with 0.04 mm^2^ square boundary) was placed at the center of each image and the investigator identified each point of intersection as overlaying a MHC I (blue), MHC IIa (red), MHC IIb (green), MHC IIx (lacking staining), or interstitium. For muscle fibers in which the topmost portion was within the boundary of the graticule, a number of muscle fibers corresponding to each fiber type were also counted. Total number of fibers counted divided by the total area sampled was calculated to determine the number of fibers per unit cross‐sectional area (number of fibers per mm^2^). Percentage of each fiber type was determined by dividing the number of fibers corresponding with each fiber type by the total number of fibers counted. Mean muscle fiber area (µm^2^) was determined by dividing the percent of tissue comprised of a particular fiber type by the appropriate fiber number per unit area.

For immunofluorescence characterization of VCAM‐1, GTN muscle sections were fixed in HistoChoice (Sigma‐Aldrich; H2904) for 45 min at room temperature, washed with phosphate‐buffered saline (PBS, 3 × 5 min), and permeabilized in 0.2% Tween‐20 for 10 min. Following wash steps (PBS, 3 × 5 min), sections were blocked for 1 hr with 10% donkey serum in PBST at room temperature. Sections were then incubated overnight (4°C) with primary antibodies against VCAM‐1 (PA5–47029 at 1:200 in PBST; ThermoFisher Scientific) and laminin (L9393 at 1:400 in PBST; Sigma‐Aldrich). Secondary antibodies (A11058 donkey anti‐goat IgG Alexa Fluor 594; ThermoFisher Scientific and 711–485–152 donkey anti‐rabbit IgG DyLight 488; Jackson ImmunoResearch; each at 1:500 in PBST) were applied for 2 hr. The sections were mounted with a coverslip using Prolong Gold antifade mountant with 4′,6‐diamidino‐2‐phenylindole (DAPI; P36931; ThermoFisher Scientific). The investigator was blinded to section identification for image capture. Midpoint of the muscle section was identified, and nonoverlapping images were captured at the sites of most area in both the lateral and medial regions of the muscle section. An overlay graticule (with 0.04 mm^2^ square boundary) was placed at the center of each image, and the investigator counted various features provided the topmost region of each feature resided within the graticule boundary. Features included muscle fibers, nodes, and nuclei. A muscle fiber was counted as VCAM‐1^+^ if any VCAM‐1 staining was apparent within the muscle fiber laminin border. Anatomical features encircled by laminin and adjacent to muscle fibers, characteristics indicative of capillaries, were classified as nodes and considered VCAM‐1^+^ when any VCAM‐1 staining was observed within the laminin border. Nuclei were counted within the sarcoplasm (central or peripheral), nodes, and interstitial regions and considered associated with VCAM‐1 when any VCAM‐1 staining colocalized with or was directly adjacent to DAPI staining. A number of muscle fibers, nodes, and nuclei were normalized by total muscle section area sampled.

### 4.5 ELISA

Muscle tissue was homogenized in PBS (25 µl per mg of tissue) containing Halt Protease Inhibitor Cocktail (Thermo Scientific, 78438). After centrifugation at 1,500 rcf for 15 min at 4°C, the supernatant was collected for ELISA analysis for cytokines (Aushon Ciraplex Cytokine 1 Array Kit; #107‐17F‐1‐AB) and growth factors (Aushon Ciraplex Custom Array Kit for VCAM‐1 and VEGF, #100‐0286) per standard kit instructions. Images of the arrays were taken using an Aushon Cirascan Imaging System. Total protein was determined using a standard colorimetric bicinchoninic acid (BCA) protein assay (Pierce, Rockford, IL, USA).

### 4.6 Statistical analysis

Data were analyzed using ANOVA (JMP version 11; SAS Institute, Inc., Cary, NC, USA) with the variable of animal identification as a random factor to account for repeated measures when appropriate. Post hoc comparisons were performed using Fisher’s least significant difference method. Correlations were assessed by Pearson product correlation analysis (SigmaPlot version 12.5; Systat Software, Inc., San Jose, CA, USA). Chi‐square analysis (SigmaPlot version 12.5) was utilized to determine training‐induced differences in absolute frequency distributions of fiber type. With the exception of frequency distribution data, all data are expressed as means ± standard error. *p* < 0.05 was considered statistically significant.

## CONFLICT OF INTEREST

The authors declare no conflict of interest.

## PUBLICATION DISCLAIMERS

The findings and conclusions in this report are those of the author(s) and do not necessarily represent the official position of the National Institute for Occupational Safety and Health, Centers for Disease Control and Prevention.

## AUTHOR CONTRIBUTIONS

E.P.R. and B.A.B. designed the study, interpreted the data, and wrote the manuscript; E.P.R., M.A.N., J.E., and B.A.B. performed the experiments. All authors approved the final version of the manuscript.
