# Supplementary material for: VCAM‐1 upregulation accompanies muscle remodeling following resistance‐type exercise in Snell dwarf (Pit1dw/dw) mice
Source: Aging Cell. 2018 Jul 10;17(5):e12816. doi: 10.1111/acel.12816 (PMC6156491; doi:10.1111/acel.12816)
Supplement: Supplementary file 1 [file ACEL-17-e12816-s001.pdf]

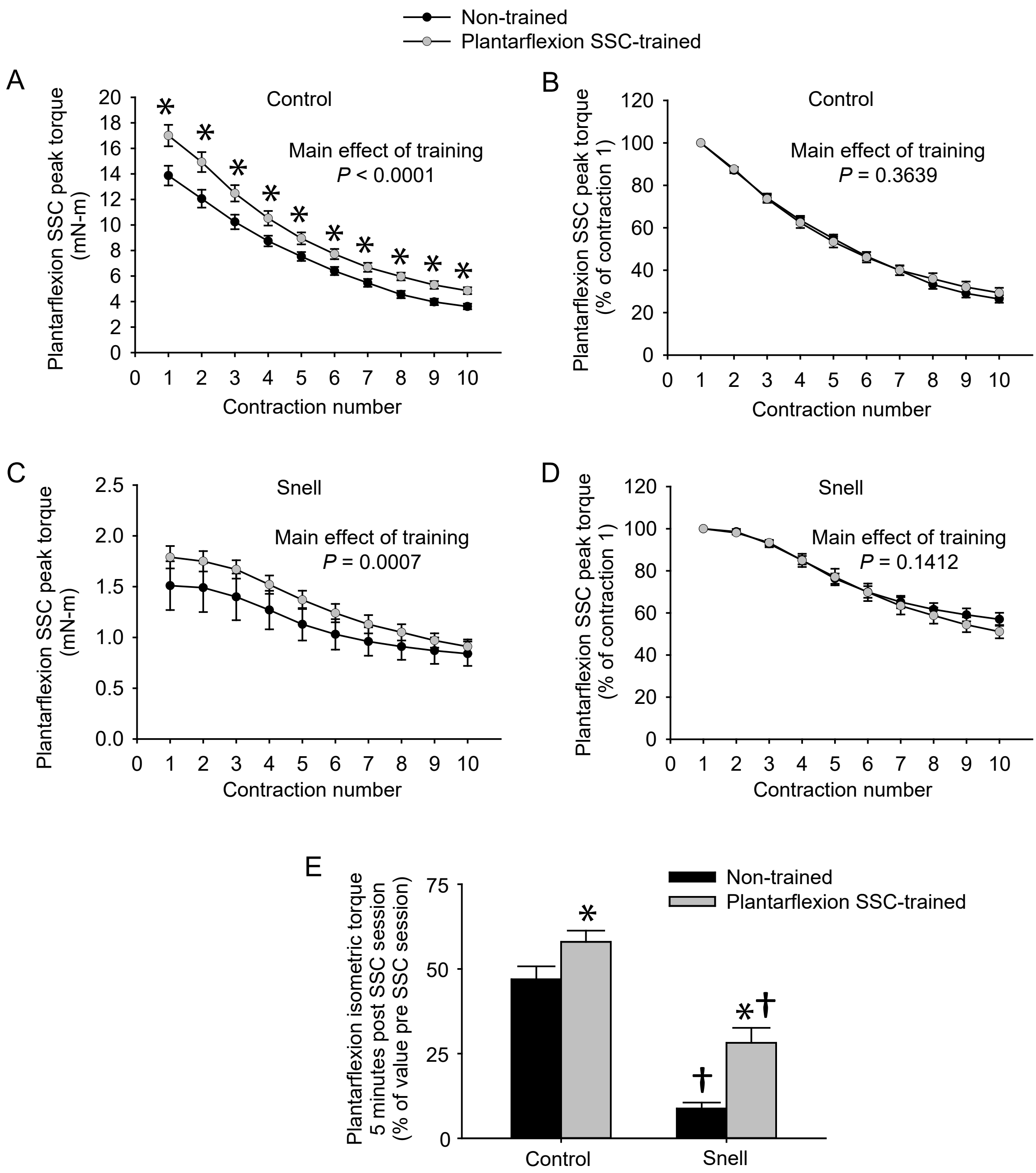

Fig. S1. Assessment of SSC-induced fatigue and recovery from fatigue. (A-D) Values for peak torque were evaluated for the first set of 10 contractions for the SSC session and expressed in absolute terms or relative to the first contraction for control and Snell dwarf mice. (E) SSC-training had a prounounced effect on recovery from fatigue especially for Snell dwarf mice as assessed by evaluating the isometric torque 5 minutes after the 80 SSC session relative to the value prior to each session. Sample sizes were *N* = 7 to 11 per group. Values are means ± SE. *Different from non-trained value, †Different from control value, *P* < 0.05.
