# Supplementary material for: VCAM‐1 upregulation accompanies muscle remodeling following resistance‐type exercise in Snell dwarf (Pit1dw/dw) mice
Source: Aging Cell. 2018 Jul 10;17(5):e12816. doi: 10.1111/acel.12816 (PMC6156491; doi:10.1111/acel.12816)
Supplement: Supplementary file 2 [file ACEL-17-e12816-s002.pdf]

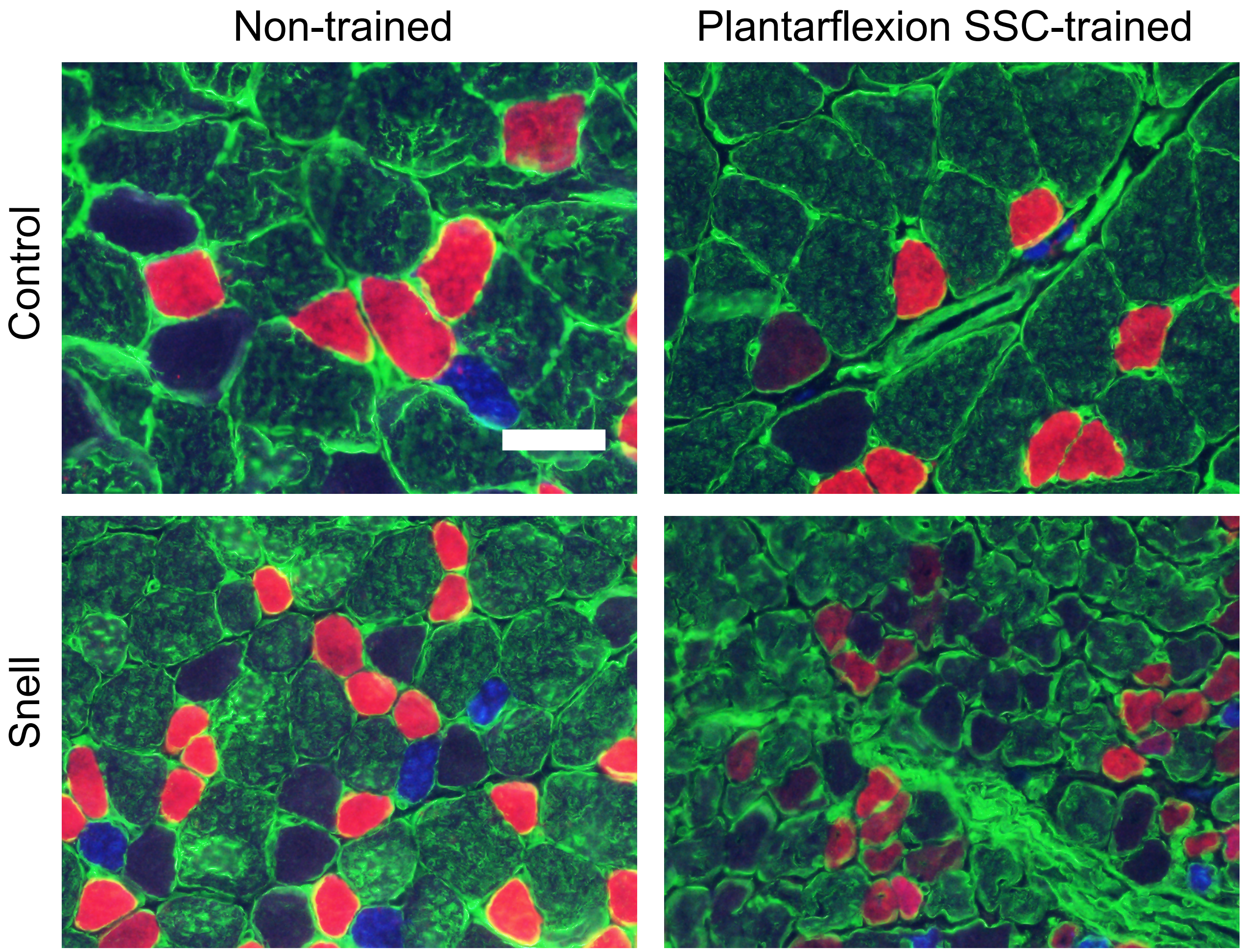

Fig. S2. An increased prevalence of type IIx fibers were apparent in GTN muscles of Snell dwarf mice following plantarflexion SSC-training. Muscle sections were analyzed by immunofluorescence staining for laminin (green) and multiple MHC isoforms - I (blue), IIa (red), IIb (green), and IIx (negative for staining). Scale bar = 50 µm.
