# Supplementary material for: VCAM‐1 upregulation accompanies muscle remodeling following resistance‐type exercise in Snell dwarf (Pit1dw/dw) mice
Source: Aging Cell. 2018 Jul 10;17(5):e12816. doi: 10.1111/acel.12816 (PMC6156491; doi:10.1111/acel.12816)
Supplement: Supplementary file 3 [file ACEL-17-e12816-s003.pdf]

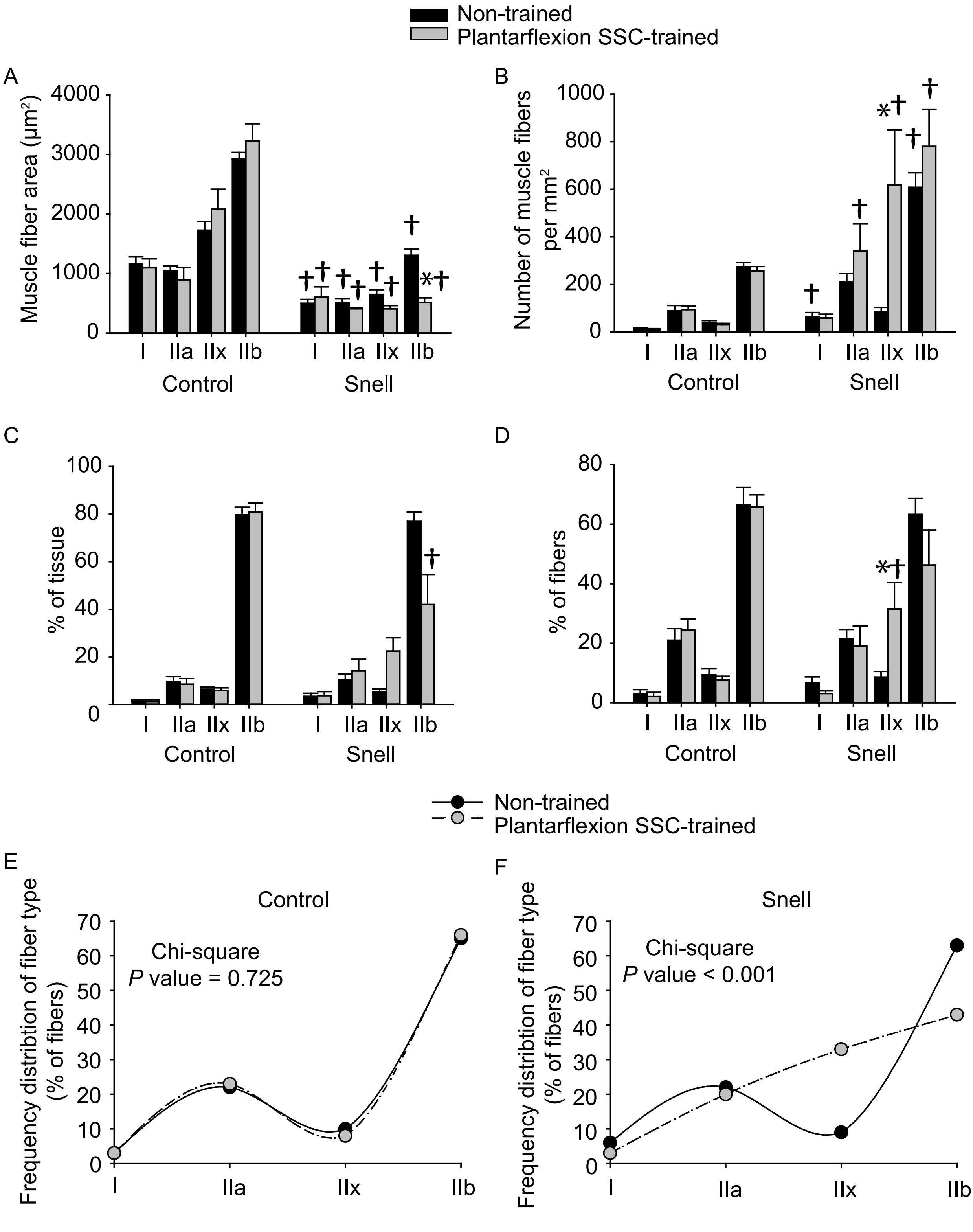

Fig. S3. Fiber type analysis demonstates a SSC training-induced increase in percentage of type IIx fibers for GTN muscles of Snell dwarf mice. (A) Muscle fiber cross-sectional areas, (B) number of muscle fibers per unit area, (C) percent of tissue composed of each fiber type, and (D) percent of total number of fibers for each fiber type expressed as means ± SE. Sample sizes were $N = 5$ to 6 per group. *Different from non-trained value, †Different from control value, $P < 0.05$. (E,F) Frequency distribution values were calculated as the total number for each fiber type across all mice of the specified group divided by the total number of all fibers for that group. Statistical differences between distributions were tested by Chi-square analysis.
