# Supplementary material for: VCAM‐1 upregulation accompanies muscle remodeling following resistance‐type exercise in Snell dwarf (Pit1dw/dw) mice
Source: Aging Cell. 2018 Jul 10;17(5):e12816. doi: 10.1111/acel.12816 (PMC6156491; doi:10.1111/acel.12816)
Supplement: Supplementary file 4 [file ACEL-17-e12816-s004.pdf]

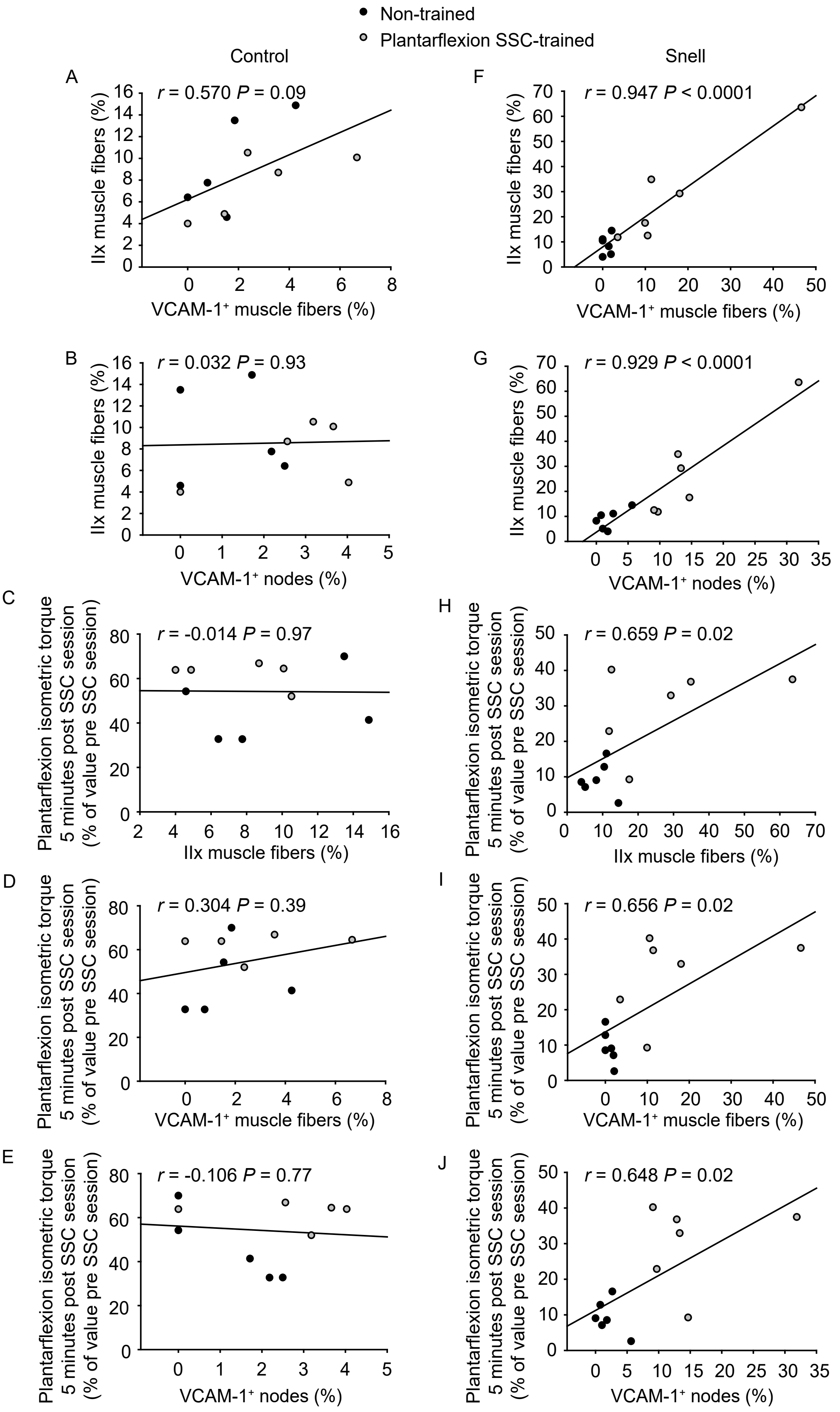

Fig. S4. A robust and coordinated remodeling response in VCAM-1 and type IIx fiber distribution unique to SSC-trained Snell dwarf mice was correlated with improved recovery from fatigue. Correlations between VCAM-1^{+} staining , type IIx fibers, and post-SSC isometric torque (a measure of fatigue recovery) were determined for (A-E) control mice and (F-J) Snell dwarf mice. Correlation coefficients and *P* values were determined by Pearson product correlation analysis. Sample sizes were *N* = 5 to 6 per group.
