# Supplementary material for: VCAM‐1 upregulation accompanies muscle remodeling following resistance‐type exercise in Snell dwarf (Pit1dw/dw) mice
Source: Aging Cell. 2018 Jul 10;17(5):e12816. doi: 10.1111/acel.12816 (PMC6156491; doi:10.1111/acel.12816)
Supplement: Supplementary file 5 [file ACEL-17-e12816-s005.docx]

Table S1. Protein levels (pg per ng total protein) for various cytokines in TA muscles.

|  |  | Non-trained | Plantarflexion SSC-trained |
| --- | --- | --- | --- |
| Control |  |  |  |
|  | IFN-ɣ | 56.2 ± 3.8 | 53.8 ± 2.9 |
|  | IL-6 | 8.8 ± 0.6 | 11.5 ± 0.7 |
|  | IL-10 | 14.8 ± 1.1 | 17.9 ± 3.5 |
|  | IL-12 | 3.6 ± 0.1 | 4.1 ± 0.2 |
|  | IL-17 | 2.2 ± 0.2 | 3.2 ± 0.2 |
|  | TNF-α | 9.4 ± 0.4 | 9.7 ± 1.3 |
| Snell |  |  |  |
|  | IFN-ɣ | 77.1 ± 7.8 | 59.7 ± 8.8 |
|  | IL-6 | 10.7 ± 1.1 | 10.4 ± 1.4 |
|  | IL-10 | 19.1 ± 1.8 | 19.2 ± 2.1 |
|  | IL-12 | 4.9 ± 0.4 | 5.6 ± 1.1† |
|  | IL-17 | 2.2 ± 0.3 | 2.2 ± 0.7 |
|  | TNF-α | 12.0 ± 1.5 | 11.0 ± 2.6 |

Values are means ± SE. Sample sizes were *N* = 3 to 9 per group. †Different from control value, *P* < 0.05.
